# Dental Law

**Published:** 1885-04

**Authors:** 


### EDITORIAL.

### Dental Law.

Below is given the text of a law to insure the better education of practitioners of Dental Surgery, and to regulate the practice of Dentistry recently enacted by the legislature of the Territory of Dakota. To this we referred in the last number of the REGISTER, but by reference now to the law itself, it will be seen that it contains the essential points of a good and efficient law. This is the first territory of our country that has made such an advance, but it is to be hoped that others, yes, all other territories and states not having such a law will soon fall into line. This law will doubtless improve the profession in Dakota, as it has done in every part of the country where such enactments have been in force. We ask especial attention to the ninth section which is a very important provision and should be embodied in every law of this kind.

*AN ACT To insure the better education of practitioners of Dental Surgery and to regulate the practice of dentistry in the Territory of Dakota.*

Be it enacted by the legislative assembly of the territory of Dakota:

Sec. 1. That it shall be unlawful for any person to engage in the practice of dentistry in this territory unless he or she shall have obtained a certificate, as hereinafter provided.

Sec. 2. A board of examiners, to consist of five practicing dentists, is hereby created, whose duty it shall be to carry out the purposes and enforce the provisions of this act. The members of said board shall be appointed by the governer, who shall select them from ten candidates, whose names shall be furnished him by the South Dakota Dental Society and the Northwestern Dental Association. Each shall furnish the names of five candidates, and the governor shall select at least two from each five names so furnished to be members of the board. The term for which the members of said board shall hold their offices shall be five years, except that the members of the board first to be appointed under this act shall hold their offices for the term of one, two, three, four, and five years respectively, and until their successors shall be duly appointed. In case of a vacancy ocurring in said board, such vacancy shall be filled by the governor from names presented to him by the Northwestern Dental Association and the South Dakota Dental Society. It shall be the duty of said dental organizations to present twice the number of names to the governor of those to be appointed.

Sec. 3. Said board shall choose one of its members president, and one the secretary thereof, and it shall meet at least once in each year, and as much oftener, and at such times and places, as it may deem necessary. A majority of said board shall at all times constitute a quorum, and the proceedings thereof shall at all reasonable times be open to public inspection.

Sec. 4. Within six months from the time this act takes effect, it shall be the duty of every person who is at that time engaged in the practice of dentistry in this territory to cause his or her name and residence, or place of business, to be registered with said board of examiners, who shall keep a book for that purpose.

The statement of every such person shall be verified under oath, before a notary public or justice of the peace, in such a manner as may be prescribed by the board of examiners.

Every person who shall so register with said board, as a practitioner of dentistry, may continue to practice the same as such, without incurring any of the liabilities or penalties provided in this act, and shall pay to the board of examiners for such registration a fee of one dollar.

It shall be the duty of the board of examiners to forward to the register of deeds of each county in the territory a certified list of the names of all persons residing in the county who have registered in accordance with the provisions of this act; and it shall be the duty of all registers of deeds to register such names in a book to be kept for that purpose.

Sec. 5. Any and all persons who shall so desire may appear before said board at any of its regular meetings, and be examined with reference to their knowledge and skill in dental surgery; and if the examination of any such person or persons shall prove satisfactory to said board, the board of examiners shall issue to such persons, as they shall find to possess the requisite qualifications, a certificate to that effect, in accordance with the provisions of this act; said board shall also indorse as satisfactory diplomas from any reputable dental college, when satisfied with the character of such institution, upon the holder of such diploma furnishing evidence, satisfactory to the board, for his or her right to the same.

All certificates issued by said board shall be signed by its officers, and such certificate shall be prima facie evidence of the right of the holder to practice dentistry in the territory of Dakota.

Sec. 6. Any person who shall violate any of the provisions of this act shall be deemed guilty of a misdemeanor, and upon conviction may be fined not less than fifty dollars nor more than one hundred dollars, or be confined six months in the county jail.

All fines received under this act shall be paid into the common school fund of the county in which such conviction takes place.

Sec. 7. In order to provide the means for carrying out and maintaining the provisions of this act, the said board of examiners may charge each person applying to or appearing before them for examinatiou for a certificate of qualification a fee of ten dollars, which fee shall in no case be returned ; and out of the funds coming into the possession of board, from the fees so charged, the members of said board may receive, as compensation, the sum of five dollars for each day actually engaged in the duties of their office, and all legitimate and necessary expenses incurred in attending the meetings of said board; said expenses shall be paid from the fees and penalties received by the board under the provisions of this act, and no part of the salary or other expenses of the board shall ever be paid out of the territorial treasury.

All moneys received in excess of said per diem allowance and other expenses, as above provided for, shall be held by the secretary of said board as a special fund for meeting expenses of said board and carrying out the provisions of this act, he giving such bonds as the board shall from time to time direct.

And said board shall make an annual report of its proceedings to the governor by the 15th of December, of each year, together with an account of all moneys received and disbursed by them pursuant to this act.

Sec. 8. Any person who shall receive a certificate of qualification from said board shall cause his or her certificate to be registered with the register of deeds of any county in which such person may desire to engage in the practice of dentistry, and the registers of the deeds of the several counties in this territory shall charge for registering such certificates a fee of twenty-five cents for such registration.

Any failure, neglect or refusal on the part of any person holding such certificate to register the same with the register of deeds, as above directed, for a period of six months, shall work a forfeiture of the certificate, and no certificate when once forfeited shall be restored, except upon the payment to said board of examiners of the sum of twenty-five dollars as a penalty for such neglect, failure, or refusal.

Sec. 9. Any person who shall knowingly and falsely claim or pretend to have or hold a certificate of license, diploma, or degree granted by any society, or who shall falsely and with intent to deceive the public, claim or pretend to be a graduate from any incorporated dental college, not being such graduate, shall be deemed guilty of a misdemeanor, and shall be liable to the same penalty as provided in section 6 of this act.

Sec. 10. This act shall take effect and be in force from and after its passage and approval.

Approved March 10th, 1885.

